# Response of upstream migrating juvenile European eel (*Anguilla anguilla*) to electric fields: Application of the marginal gains concept to fish screening

**DOI:** 10.1371/journal.pone.0270573

**Published:** 2022-06-30

**Authors:** Mhairi Miller, Suleiman M. Sharkh, Paul S. Kemp

**Affiliations:** International Centre for Ecohydraulics Research, Faculty of Engineering and Physical Science, University of Southampton, Southampton, United Kingdom; Swedish University of Agricultural Science, SWEDEN

## Abstract

The decline in European eel (*Anguilla anguilla*) recruitment over the past half-century is partly due to river infrastructure that delays or blocks upstream migration to rearing habitat. Stimuli, such as electricity, can be used to modify the behaviour of downstream moving fish and guide them to preferred routes of passage at river infrastructure; but research on upstream migrating juvenile eel remains limited. The response of upstream migrating juvenile eel exposed to pulsed direct current (PDC) electric fields was investigated using a recirculatory flume. Eel were presented a choice of two routes upstream under either: (1) a treatment condition, in which the selection of one route resulted in exposure to High Electric Field (HEF) strength that was between 1.5–2 times stronger than the Low Electric Field (LEF) strength encountered in the alternative route; or (2) a control in which the electric field was absent in both routes. Under the treatment, five different mean HEF strengths (0.53, 0.77, 1.22, 2.17 and 3.74 Vcm^-1^) were tested at one of two frequencies (2 and 10 Hz). Route choice, distance downstream of the first set of electrodes at which an initial response was observed and avoidance behaviours (*acceleration*, *retraction*, *switching* and *rejection*) were compared among treatments. For the 1.22, 2.17 and 3.74 Vcm^-1^ and under 2 Hz, eel preferred to pass the LEF route. Avoidance was greater in the HEF route and positively related to field strength. The distance of the initial response did not differ between routes, field strengths or frequency. Upstream migrating eel avoided electric fields indicating potential to develop this approach for fish guidance. Further work is needed to test prototypes in field settings, particularly in combination with traditional physical screens to water intakes as part of a process of applying the concept of marginal gains to advance environmental impact mitigation technology.

## 1. Introduction

The catadromous European eel (*Anguilla anguilla*) has experienced substantial declines in escapement and recruitment since the 1970s [1]. Juvenile (glass) eel recruitment has reduced by more than 90% in some catchments (e.g. River Thames; [2]), with this life-stage representing an important population bottleneck [2, 3]. A decline in recruitment is translated to a reduction in eel density in freshwater habitats and ultimately lower spawning escapement of adults [4]. As both the juvenile and adult life-stages have historically maintained fisheries of high commercial importance [4, 5], legislation has been enacted (e.g. Eel Regulation, European Council Regulation 1100/2007) to promote sustainable management and aid recovery across its range [6, 7].

There are several potential causes for the decline of European eel, including pollution [8], habitat loss [9], overfishing [10], and non-native parasites [11, 12]. In the estuarine and freshwater environment, anthropogenic structures (e.g. barrages, dams, and weirs) can block or delay both the downstream adult (e.g. [13, 14]) and upstream juvenile migration (e.g. [15–18]), representing a substantial challenge to escapement and recruitment [9]. River infrastructure can also cause direct mortality of juvenile and adult eel entrained into water intakes, e.g. at hydroelectric or thermal power plants [4, 19]. As eel are entrained into water intakes to abstraction points, mortality can occur due to sharp changes in water temperature (e.g. in cooling water systems) and pressure, and mechanical damage caused by striking moving parts [19–22]. Historically, research has been biased to the risks of infrastructure faced by downstream moving adults, while the threats to upstream migrating juvenile eel are less well understood [23]. Mitigating the impacts of river infrastructure on juvenile eel provides a feasible and important management option that can be adopted in the estuarine and freshwater domain.

Traditionally focusing on the adult life-stage, physical screens reduce or prevent the passage of eel into water intakes. However, these are not wholly effective and can incur high construction and maintenance costs [21, 24]. Due to their small size, juvenile eel are unlikely to be blocked by existing screens designed for larger target species and life-stages and hence require expensive retrofits with very narrow-spaced designs (1–2 mm) [25]. Furthermore, eel may be impinged on poorly designed screens and suffocate if they are unable to escape because the velocities at the screen face exceed their burst swimming capabilities [21, 24]. Even if impinged eel can escape, like other species of fish (e.g. [26] for delta smelt, *Hypomesus transpacificus*), they are likely to suffer physical injury due to the abrasion experienced when contacting the screen surface, resulting in secondary infection and delayed mortality.

Behavioural deterrents employed in fish guidance and exclusion, such as those based on acoustics [27, 28], bubbles [29, 30], light [31] and electricity [32], have been developed as an alternative to traditional physical screens. They have the advantage of not requiring physical or mechanical elements, and thus lack the potential to cause impingement and abrasion. Unfortunately, they tend to be less effective than physical screens, resulting in being promoted by regulatory agencies only when physical exclusion screens are impractical [33]. However, behavioural deterrents may have an important role to play when used in conjunction with physical screens to reduce the negative impacts of the latter and improve overall system efficiency. For example, under experimental conditions acoustic stimuli have been used to enhance the effectiveness of physical screens in guiding downstream moving eel [34]. This approach is based on applying the concept of Marginal Gains to advance environmental impact mitigation technology. Originally developed in the field of performance sport (e.g. [35]), the principle of Marginal Gains is that small incremental improvements in any process amount to a significant improvement when considering the system holistically. One of the main advantages of this approach in this context is that if the behavioural deterrents work then they can provide a cost-effective addition to physical and mechanical screening systems, with relatively low capital and maintenance expense compared to fine-screen retrofits [33, 36]. For eel, acoustics [28, 34, 37] and light [38, 39] have garnered most interest, indicating variable efficacy, while electricity has received limited attention (e.g. [40]).

Designing a suitable electric deterrent for a target species requires testing of the most effective field characteristics (e.g. field strength and pulse frequency). For example, a field strength that is too weak would be ineffective, while one that is too strong may stun the fish, rendering it incapable of exhibiting the voluntary response needed, or even worse, injuring or killing it due to an excessive electric shock. The use of electric fields is likely to be more effective in the guidance of fish moving in the upstream, rather than downstream, direction. Should an individual be shocked and temporarily paralysed by an ill designed device, an upstream moving fish will be swept downstream away from the field where it should recover [41]. Conversely, an incapacitated downstream moving fish would be swept into the field and possibly into the dangerous area (e.g. turbine intake) that the device was designed to screen. Pulse frequency can also influence fish behaviour. For example, greater avoidance (i.e. failure to cross an electric field array) to an electric barrier was observed under a 3 Hz as opposed to 2 Hz pulse frequency for Pacific salmon steelhead [*Oncorhynchus mykiss*] and Pacific lampreys [*Entosphenus tridentatus*] [42]. However, while increasing frequency might be beneficial for effectively deterring fish movement, other studies suggest that more injuries can occur as frequency increases (e.g. silver carp; [43]).

This study investigated the response of upstream migrating juvenile (glass) eel to pulsed direct current (PDC) electric fields when offered a choice of route in an experimental flume. In the test section, the flume was divided longitudinally into two routes of equal dimension by a series of eight earthed vertical electrodes, presenting the upstream swimming eel with an opportunity to select a route: (1) of differing field strength (High or Low Electric Field Strength–HEF / LEF) under the treatment conditions; or (2) a control in which the electric field was absent in both routes. The influence of electric field strength and frequency on: (1) route selection, (2) distance from the electrodes at which an initial response was exhibited by upstream moving juvenile eel, and (3) nature (*acceleration*, *retraction*, *switching* and *rejection*) of avoidance response was investigated. We predicted that: (1) eel would prefer (deviation from the 50:50 route selection expected if choice was random) to pass the LEF route when offered a choice; and (2) distance of the initial response from the source of the EF and (3) occurrence of avoidance behaviour exhibited would be positively related to field strength, and that these relationships would be stronger under the high frequency (10 Hz) condition.

## 2. Materials and methods

### 2.1 Ethical note

This study was performed after approval from the University of Southampton’s Ethics and Research Governance Office (Ethics ID 46983). After experimental trials all eel were released back into the wild as requested by the Environment Agency.

### 2.2 Experimental set-up

**Fig 1:**
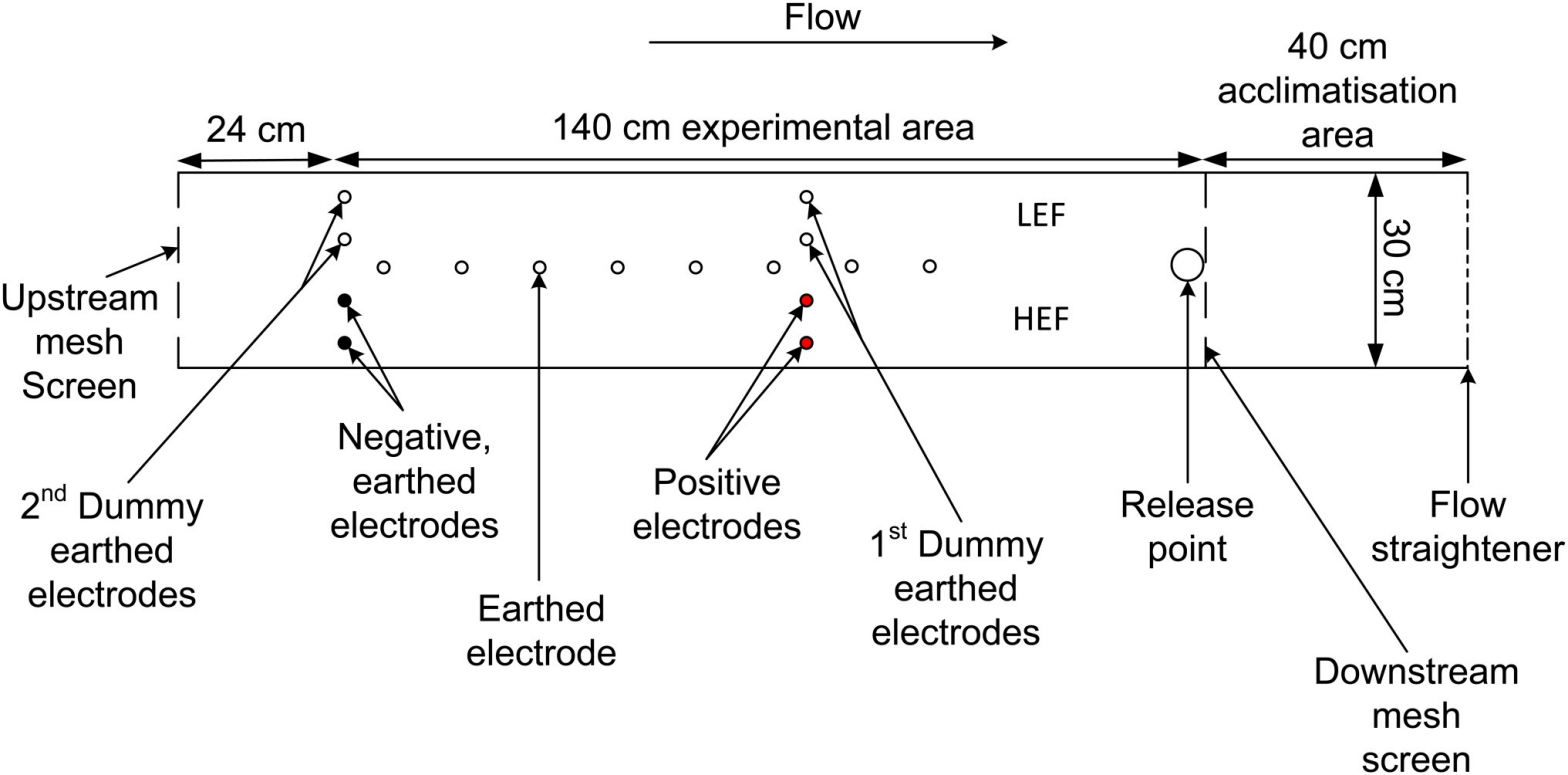
Experimental set-up used to investigate route choice of upstream moving juvenile European eel in response to encountering electric fields of differing strength. The flume was divided by eight centrally placed earthed electrodes separated 12 cm apart. Two positive and two negative earthed electrodes were installed to create a High Electric Field (HEF) strength route along one side of the channel. An adjacent Low EF (LEF) strength route was created by having two sets of two earthed electrodes (1^st^ and 2^nd^ dummy earthed electrodes, respectively) at the same longitudinal position as the positive and negative earthed electrodes in the HEF.

The experiment was conducted in an indoor open channel flume (12.0 m long, 0.3 m wide and 0.4 m deep). Within the flume a 2.04 m long section was isolated by a downstream flow straightener and upstream plastic mesh screen (mesh size: 0.28 x 0.79 mm) (Fig 1). Eight earthed cylindrical steel electrodes (80 cm long x 1 cm diameter) were installed longitudinally down the centre of the flume at 12 cm intervals, dividing the channel into two routes that under the treatment conditions were defined as either High (HEF) or Low Electric Field (LEF) strength. In the HEF route, two positive and two negative earthed electrodes were installed laterally at 6 cm intervals (Fig 1). The LEF route had two sets of two earthed electrodes (1^st^ and 2^nd^ dummy earthed electrodes, respectively) at the same longitudinal position as the positive and negative earthed electrodes in the HEF route. Earthed electrodes were arranged to best localise the electric field to the HEF route and prevent the field extending outside the experimental area. Each electrode was covered and secured with mesh fabric (mesh size: 0.28 x 0.79 mm) to prevent direct contact by the eel.

A black screen was placed alongside the flume to prevent disturbance to the fish by the observers. Six CCTV cameras (SWANN 1080p; 1920 X 1080 pixel resolution) were positioned above the experimental area (55 cm above the base of the flume) along the length of the flume. Two infrared lights (780–850 nm wavelength, assumed non-visible to glass eel) were positioned at the downstream and upstream mesh to provide sufficient illumination for video capture under conditions of darkness.

Ambient light levels measured prior to the start of each trial were consistently less than 0.01 lux. Water depth and velocities were measured (Valeport Model 801) upstream of the last set of electrodes, within the electrode array (i.e. halfway longitudinally between the first and last set of electrodes), and downstream of the first set of electrodes after every five trials (Upstream: mean [± SE] = 18.5 [± 0.10] cm and 0.121 [± 0.0009] ms^-1^; Electrode Array: 18.4 [± 0.11] cm and 0.135 [± 0.001] ms^-1^; Downstream: 17.8 [± 0.15] cm and 0.118 [± 0.001] ms^-1^). The velocities were measured midway in the water column and at three lateral points across the flume. The test velocities were selected based on juvenile eel prolonged swimming capabilities of 0.2–0.4 ms^-1^ and maximum burst speeds of up to 0.5 ms^-1^ [44]. This ensured that responses observed were a result of the electric field and not influenced by their swimming capabilities.

**Fig 2:**
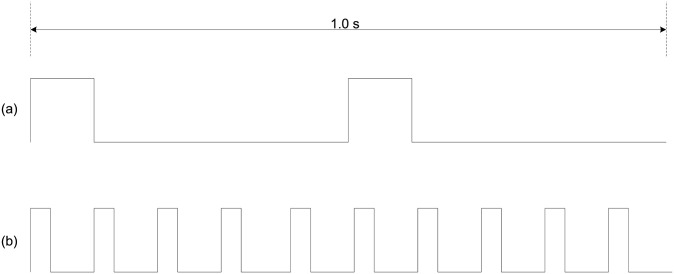
PDC frequencies (a: 2 Hz; b: 10 Hz) used to investigate juvenile eel response to electric fields in flowing water.

A Smith-Root Electrofishing pulse generator (BP-1.5 POW) was used to generate five different electric field strengths in the HEF route (Table 1) based on pilot testing of a subset of eel (n = 38). Two different pulse frequency PDC waveforms were implemented: (a) 2 Hz (100 ms pulse width) and (b) 10 Hz (20 ms pulse width) (Fig 2). PDC waveforms with these pulse frequencies were selected based on evidence that frequencies < 15 Hz reduce injuries in eel [45] while providing an effective deterrent in other species (e.g. 2 Hz: white sturgeon, *Acipenser transmontanus*, [46]; 10 Hz: fathead minnows, *Pimephales promelas*, [47]). Pulse widths were generated by maintaining the same duty cycle (20%) between frequencies. Hence, in total there were 10 treatment conditions and one control (Table 1).

**Table 1:** The characteristics of the electric fields generated in adjacent High (HEF) and Low Electric Field (LEF) strength routes in an experimental flume under 10 treatments and a control.

Pulse Generator Output (V)	High Electric Field (HEF) Strength (Vcm^-1^)		Low Electric Field (LEF) Strength (Vcm^-1^)		Replicates (n)
	Mean [± SE]	Range	Mean [± SE]	Range	
0 (Control)	0	0	0	0	21
10	0.53 [± 0.06]	0–1.48	0.27 [± 0.03]	0–1.11	2 Hz: 25
					10 Hz: 25
15	0.77 [± 0.08]	0–2.22	0.44 [± 0.04]	0–1.48	2 Hz: 23
					10 Hz: 25
20	1.22 [± 0.13]	0–3.70	0.71 [± 0.06]	0–2.59	2 Hz: 23
					10 Hz: 25
25	2.17 [± 0.22]	0–5.56	1.28 [± 0.14]	0–4.44	2 Hz: 24
					10 Hz: 22
30	3.74 [± 0.34]	0–9.26	2.42 [± 0.22]	0–7.41	2 Hz: 26
					10 Hz: 24

The experiment investigated the influence of electric field strength on route choice of upstream moving juvenile European eel. The number of replicates (n) for each treatment is provided. Note the High/ Low Electric field strengths were equivalent under both 2 and 10 Hz.

**Fig 3:**
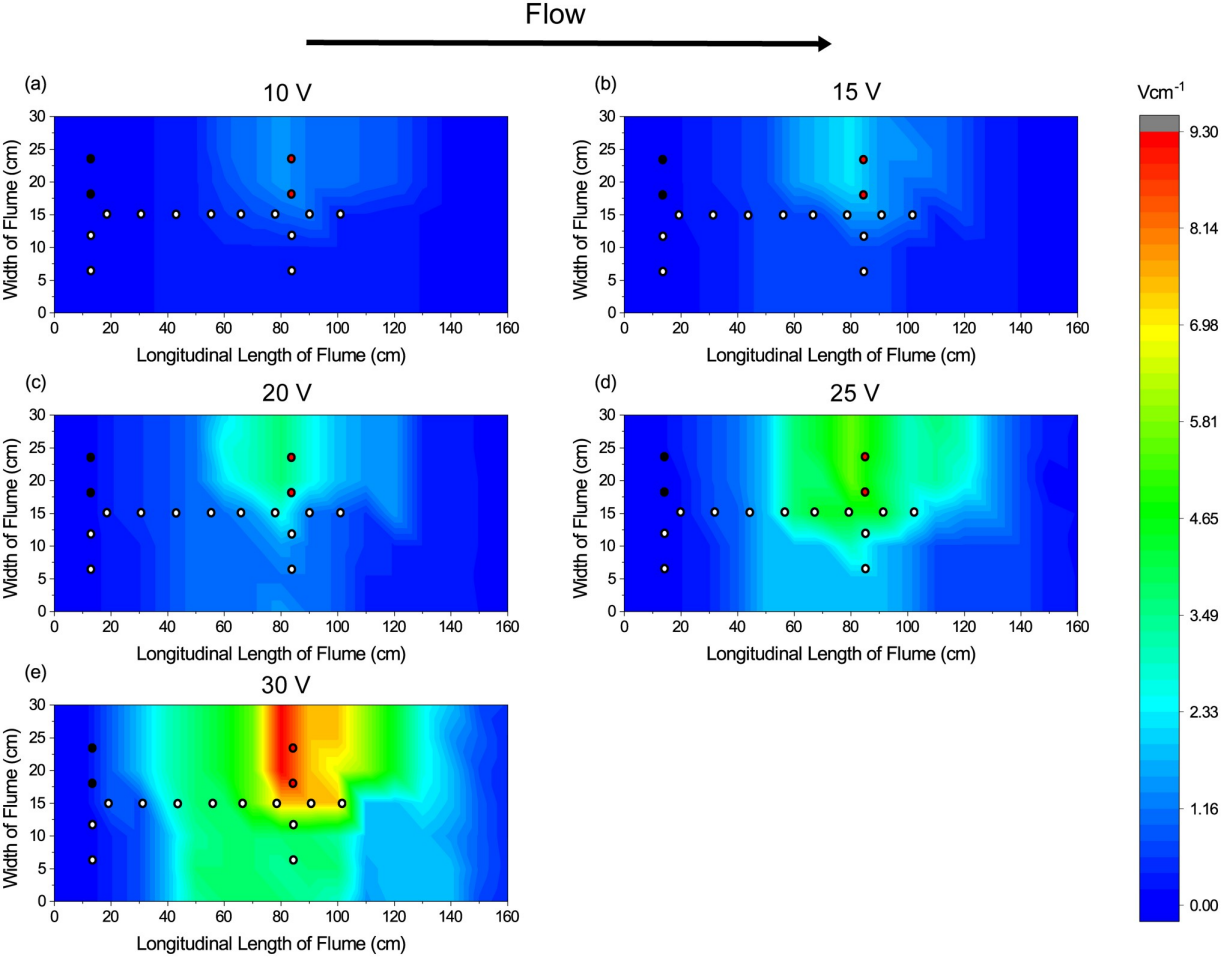
Electric field (Vcm^-1^) generated by the pulse generator output of (maps represent both 2 and 10 Hz frequencies as field distribution was the same): (a) 10 V (Mean HEF Strength: 0.53 Vcm^-1^), (b) 15 V (Mean HEF Strength: 0.77 Vcm^-1^), (c) 20 V (Mean HEF Strength:1.22 Vcm^-1^), (d) 25 V (Mean HEF Strength: 2.17 Vcm^-1^) and (e) 30 V (Mean HEF Strength: 3.74 Vcm^-1^). The *x* axis represent the longitudinal distance along the flume and the *y* axis the width of the flume. Centrally earthed electrodes (white dots) were positioned at *x* = 18, 30, 42, 54, 66, 78, 90, and 102 and at *y* = 15. Positive electrodes (red dots) were positioned at *x* = 84 and *y* = 18 and 24. Negative earthed electrodes (black dots) were positioned at *x* = 12 and *y* = 18 and 24. Dummy earthed electrodes (white dots) were at (84, 6) and (84, 12) and (12, 6) and (12, 12). Measurements were taken at 10 cm water depth from the surface.

The electric field was mapped using a potential probe comprising two-point conductors 27 mm apart connected to an oscilloscope (Gwinstek GDS-1052-U) via a differential probe module (Probemaster 4232). Measurements were taken in a grid at a spacing of 10 cm in the *x* and 5 cm in the *y* directions at a water depth of 10 cm (from the surface) to record peak-to-peak voltage (Fig 3). Field strength was calculated as the quotient of the peak-to-peak voltage and the distance between the two-point conductors. Maps of the electric field were created for all five HEF strengths, under both the 2 and 10 Hz pulse frequencies (Fig 3). Water conductivity was 580 μS.

### 2.3 Fish husbandry

Glass eel (batch of 200 g) were captured from the River Severn by UK Glass Eel Ltd on 25 February 2019 and transported in chilled river water (8°C) to the International Centre for Ecohydraulics Research laboratory at the University of Southampton. Fish were held in a porous container (for between 2–11 days) in the sump of the flume where the water was chilled to 8°C at the time of their arrival and increased gradually by 2°C daily until a target temperature of 12°C was reached. Daily sump temperatures were recorded manually and with submersible temperature loggers (mean holding sump acclimation temperature [± SE] = 12.5 [± 0.04]°C). Two aquarium air pumps were used to provide aeration. Fish health and water quality was monitored daily to ensure consistent conditions (pH: 7.8–8.4, Ammonia: 0 ppm, Nitrite: 0 ppm, Nitrate: < 40 ppm).

### 2.4 Experimental procedure

Trials were conducted during hours of darkness (18:00–02:00), to replicate the natural nocturnal migration of glass eel [48], between 27 February and 8 March, 2019.

A single eel was removed from the holding tank and placed in a plastic tube secured at both ends with mesh coverings before being placed in a 0.4 m long acclimatisation zone located at the downstream end of the flume section (Fig 1). Eel were allowed a minimum of 60 minutes to acclimatise, before being released centrally immediately upstream of the downstream mesh that separated the acclimatisation zone from the experimental area (release point, Fig 1). Each trial lasted a maximum of 60 minutes or until the eel had passed through the final (most upstream) set of electrodes. Each eel was used once only. Treatment electric field strengths and pulse frequencies were alternated across trials in a systematic design and the side of the flume assigned HEF / LEF was switched daily to prevent side bias and ensure even spread of treatments.

Flume temperature was maintained close to the target of 12°C (mean [± SE] = 12.6 [± 0.01]°C), as the migratory behaviour of glass eel is reported to decline below a threshold temperature range of around 11–12°C [49–51], and trials were terminated if the temperature exceeded 13°C. Water conductivity (HANNA HI98303 Conductivity Meter; mean [± SE] = 582.8 [± 0.33] μS) and eel length (mean total length [± SE] = 7.04 [± 0.019] cm) and mass (mean body mass [± SE] = 0.42 [± 0.006] g) was measured at the start and end of each trial, respectively.

### 2.5 Fish behaviour

Analysis of video recordings allowed the characterisation and quantification of route choice and avoidance exhibited (Table 2) as eel passed through the experimental area (Fig 1). Behaviour was recorded during the entire trial, and in the event that an eel exhibited more than one response type it was assigned that of the highest magnitude (1–5) (hierarchy of response adapted from [52], Table 2).

**Table 2:** Definitions of different categories of avoidance behaviours exhibited by upstream migrating juvenile eel on encountering an electric field in a flume.

Metric	Definition
(5) *Rejection*	180° turn in body position and downstream movement for at least one body length
(4) *Switching*	Movement from one route (HEF/LEF) to the other
(3) *Retraction*	Recoil of body in direction of travel of at least half body length
(2) *Acceleration*	Increase in swimming speed
(1) *No change*	No change in swimming speed or body orientation

The response is ranked in a hierarchy of magnitude from 1 (lowest) to 5 (highest) (Adapted from [52]).

Image analysis software (Logger Pro v. 3.8.2, Vernier Software) was used to obtain *x* and *y* spatial coordinates for the downstream distance (cm) from the positive electrodes in the HEF and the first dummy earthed electrodes in the LEF at which the initial response (any of the defined behaviours in Table 2 observed in the experimental area) occurred.

### 2.6 Statistical analyses

Statistical analyses were conducted using R 4.0.2 [53]. Normality was assessed using the Shapiro-Wilk test. Attempts were made to transform data to achieve normal distributions, and if unsuccessful, non-parametric tests were performed. A goodness-of-fit (χ^2^) test determined whether the route choice (HEF or LEF) deviated from the 50:50 ratio expected if the selection was random (null hypothesis–equal probability of selecting either channel under both control and treatment conditions). For statistical purposes the route that was assigned the HEF during the treatment conditions was also designated as the HEF for the control, even though the electric field was absent. Due to the low number of observations of some of the behaviours (i.e. *rejection*), all defined responses (*acceleration*, *switching*, *retraction* and *rejection*) were combined as a single avoidance response. Differences between the routes, field strength and frequency for distance of initial response were analysed using separate Kruskal-Wallis tests. A generalised linear mixed model (GZLMM) with a binomial distribution was used to determine whether route choice (HEF or LEF), field strength or frequency influenced avoidance behaviour. Eel-ID was included as a random effect as some individuals sampled both routes (HEF and LEF) and so have two observations.

## 3. Results

### 3.1 Route choice

**Fig 4:**
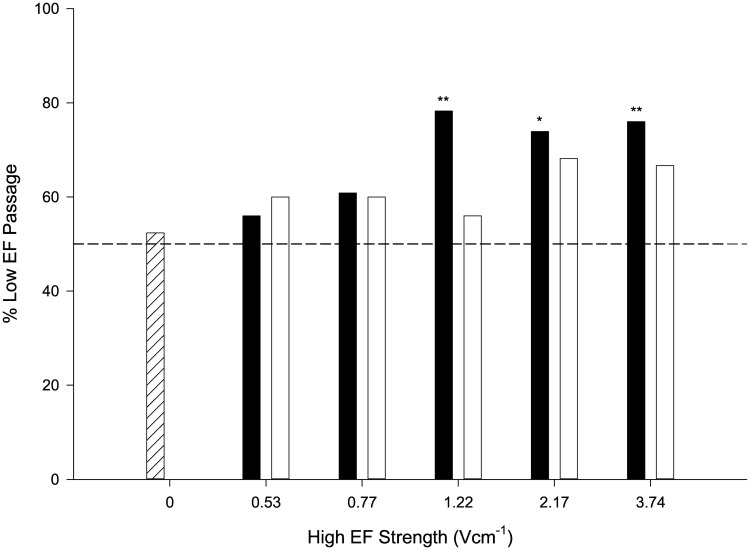
Percentage of upstream migrating juvenile eel that passed the Low Electric Field (LEF) route under the five High EF strengths (0.53, 0.77, 1.22, 2.17 and 3.74 Vcm^-1^) for both 2 Hz (solid bars) and 10 Hz (clear bars) treatments. Control (0 Vcm^-1^) is shown as white hatched bar. The dashed line indicates the expected (50% frequency) random selection if electric field strength has no influence on route choice. Note * denotes p < 0.05 and ** p < 0.01.

The percentage of eel that passed through the LEF route did not differ from 50% under the 0 Vcm^-1^ control, 0.53 Vcm^-1^ and 0.77 Vcm^-1^ treatments (p > 0.05) (Fig 4). There was also no deviation from 50% LEF passage in the 10 Hz treatment for 1.22, 2.17 and 3.74 Vcm^-1^ (p > 0.05) (Fig 4). Conversely, more eel passed through the LEF route in the 2 Hz treatment for 1.22, 2.17 and 3.74 Vcm^-1^ with a significant deviation from 50% (1.22 Vcm^-1^: χ ^2^(1) = 7.35, p = 0.007, 2.17 Vcm^-1^: χ^2^(1) = 5.26, p = 0.02 and 3.74 Vcm^-1^: χ ^2^(1) = 6.76, p = 0.009) (Fig 4).

### 3.2 Distance of initial response

**Fig 5:**
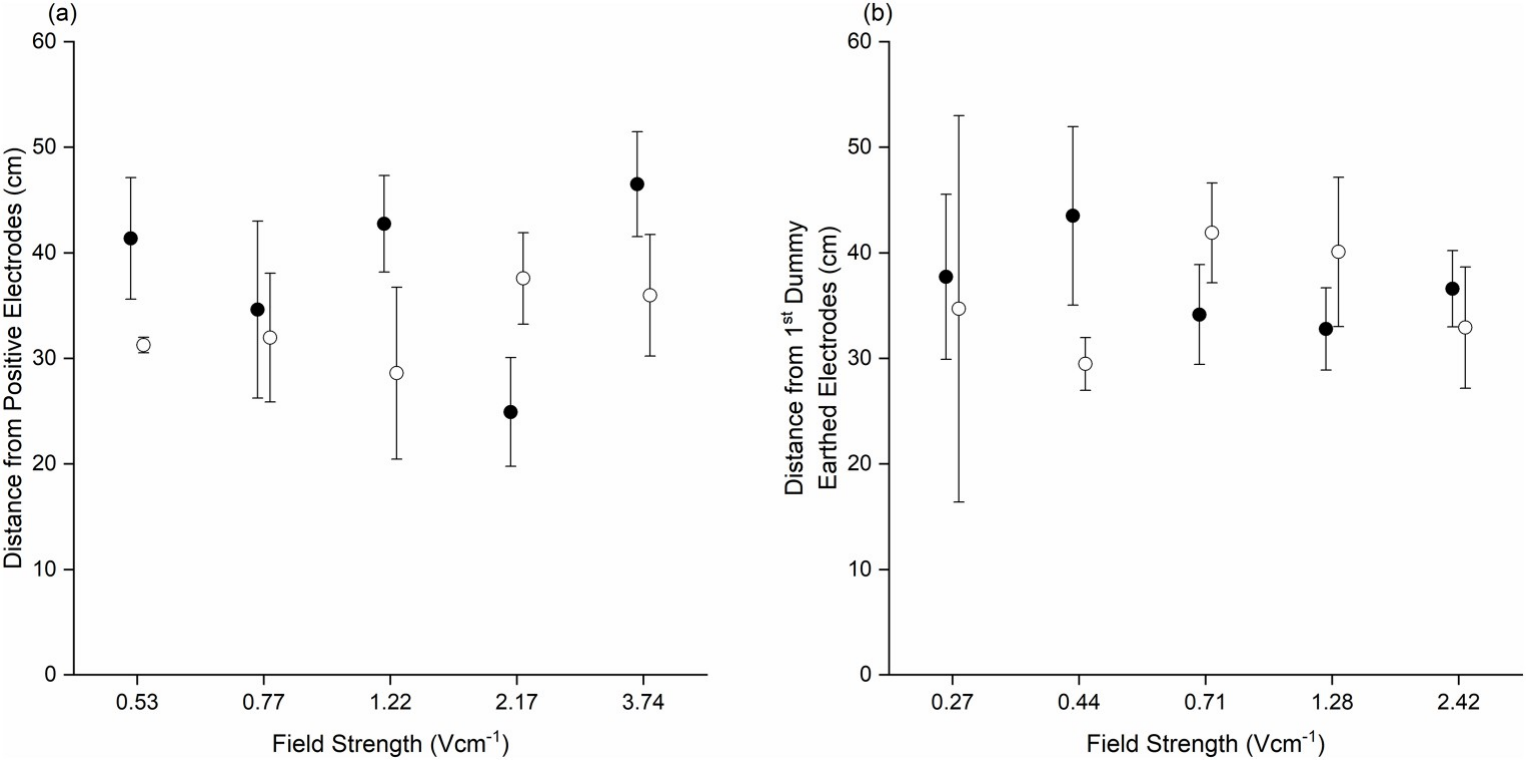
Distance (cm) of initial response [± SE] from positive and 1^st^ dummy earthed electrodes in both (a) HEF and (b) LEF route, respectively, for upstream migrating juvenile eel in the 2 Hz (solid circles) and 10 Hz (clear circles) treatment.

Distance of initial response was assessed only for those that occurred downstream of the positive electrodes, any response that occurred upstream was omitted (n = 7). Route had no effect on the distance of initial response (χ ^2^(1) = 0.39, p = 0.53). Consequently, data was aggregated for comparison between field strength and frequency. There was no effect of field strength (χ ^2^(4) = 1.1, p = 0.89) or frequency (χ ^2^(1) = 0.61, p = 0.44) on the distance of initial response (Fig 5).

### 3.3 Avoidance behaviour

**Fig 6:**
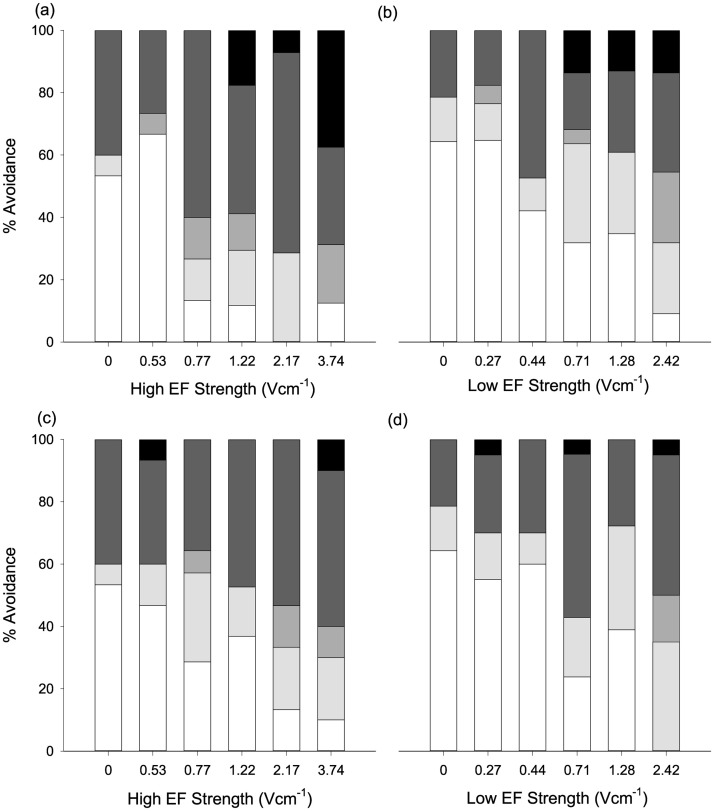
Influence of field strength and pulse frequency on the avoidance response exhibited by upstream moving juvenile eel in the HEF or LEF route. (a) HEF, 2 Hz, (b) LEF, 2 Hz, (c) HEF, 10 Hz and (d) LEF, 10 Hz. Clear, light grey, mid grey, dark grey and solid bars correspond to *no change*, *acceleration*, *retraction*, *switching* and *rejection*, respectively.

The exhibition of avoidance differed with route selected (χ ^5^^2^(1) = 4.75, p = 0.03) with more eel responding in the HEF (73.7%) than the LEF (63.0%) route (Fig 6). The most common behaviour exhibited in the HEF was *switching* (44.0%), whereas *no change* was more frequent in the LEF route (37.0%).

As avoidance differed between routes, the results in the HEF and LEF were analysed separately to enable comparisons between field strength and frequency. For the HEF, behaviour was influenced by field strength (χ ^2^(5) = 25.5, p < 0.001), with eel more likely to exhibit avoidance under 2.17 Vcm^-1^ (93.1%, z = 3.05, p = 0.03) and 3.74 Vcm^-1^ (88.9%, z = 2.99, p = 0.03) than the control (46.7%). There was also greater avoidance in the 2.17 Vcm^-1^ (z = 3.5, p = 0.006) and 3.74 Vcm^-1^ (z = 3.63, p = 0.004) than the 0.53 Vcm^-1^ (43.3%) treatment. Avoidance was not affected by pulse frequency (χ ^2^(1) = 0.72, p = 0.4). The percentage of eel exhibiting *rejection* in the HEF was < 10% for all field strengths except for 3.74 Vcm^-1^ (22.2%). Field strength was also influential in the LEF route, (χ ^2^(5) = 25.9, p < 0.001), with eel more likely to exhibit avoidance under 2.42 Vcm^-1^ (95.2%, z = 3.92, p = 0.001) than the control (35.7%), 0.27 Vcm^-1^ (40.5%, z = 4.23, p < 0.001), 0.44 Vcm^-1^ (48.7%, z = 3.85, p = 0.0015) and 1.28 Vcm^-1^ (63.4%, z = 3.08, p = 0.02) treatments. For all the field strength treatments in the LEF the percentage of eel exhibiting *rejection* was < 10%. Avoidance was not influenced by pulse frequency (χ ^2^(1) = 0.004, p = 0.95).

## 4. Discussion

Under the experimental conditions described, upstream migrating juvenile European eel (*Anguilla anguilla*) were more likely to pass a route in which they encountered a weak electric field (Low Electric Field [LEF] strength) than one with a strong field (High Electric Field [HEF] strength) when offered a choice, but only at the higher field strengths (1.22, 2.17 and 3.74 Vcm^-1^) and under the 2 Hz frequency treatment. More eel exhibited avoidance in the HEF route, and avoidance was positively related to field strength. There was no relationship between distance of initial response and field strength or frequency. To the best of our knowledge, this is the first study to directly test guidance and avoidance in juvenile (glass) eel in relation to PDC electric fields. The results support the potential use of electric fields as a method for guiding juvenile European eel movements, perhaps in combination with traditional physical screens at intakes to abstraction points as part of the application of the marginal gains concept to advancing environmental impact mitigation technology [34].

Eel were more likely to pass the LEF route under the higher field strengths (1.22, 2.17 and 3.74 Vcm^-1^), suggesting that there is some threshold at which guidance is induced. Interestingly, this was the case only under 2 Hz treatment, contradicting the prediction greater avoidance occurs at higher pulse frequencies [42]. A possible explanation for the observations is that the longer pulse width (100 ms for the 2 Hz treatment compared to 20 ms for the 10 Hz waveform) may have resulted in a higher mean power transmitted to the fish due to an exponential rise in current under the lower frequency condition [54]. In the 2 Hz waveform treatment the current has time to rise to a maximum and fall back to zero during each period; a process that is less likely to occur in the 10 Hz waveform treatment due to the higher density of pulses. If this was the case, eel would have experienced a greater variation in current when encountering a 2 Hz waveform, potentially explaining a greater influence on behaviour despite a higher frequency of pulses in the 10 Hz treatment. Furthermore, a longer ‘off’ period between pulses in the 2 Hz treatment may have limited the possibility of acquired insensitivity to the stimulus over time (e.g. due to increased tolerance or habituation that has been found for acoustic cues with shorter intervals between stimuli e.g. [55]). Interestingly, our findings support the observations for different families of fish (e.g. juvenile and adult rainbow trout, *Oncorhynchus mykiss*) in that longer pulse widths can enhance the efficiency of experimental electric deterrents [56]. These observations have important implications for the design of effective electrical deterrents that do not negatively impact fish welfare, as injury and mortality is positively related to pulse frequency (e.g., [43, 57–61]).

Although the distance of the initial response did not differ between route, field strength or frequency, when given a choice eel avoidance was higher in the HEF route, and overall avoidance in both channels increased with the strength of the electric field. This is not unexpected considering that the voltage gradient across the fish (anterior to posterior if actively swimming upstream) is a strong predictor of fish response to electric fields [62]. When pulsed (i.e. PDC), the electric field alternates between an on and off phase, and if very high field strengths (i.e. higher than those used in this study) are used the rapid change in the voltage gradient across the fish body can elicit extreme responses, such as muscular convulsions and possible spinal injury [63]. In the HEF route and under the higher field strengths, the rate of change in voltage gradient across the body between the on and off periods was greater than for the alternative treatments, resulting in an elevated probability of avoidance. Studies that explored the relationship between injury and electric fishing (e.g. [64] for Yellowstone cutthroat trout, *Oncorhynchus clarkii bouvieri*, and rainbow trout) and invasive species management (e.g. [65] for rainbow trout embryos), rather than guidance, also reported greater mortality at higher voltages.

While some avoidance behaviours, such as *acceleration*, *switching*, *retraction* were exhibited consistently in all treatments, higher field strengths were required to elicit *rejection* (i.e. a movement in the opposite direction). Moreover, relatively high rates of *rejection* (> 10% of eel) were observed in the HEF route under the 3.74 Vcm^-1^ only. This might be explained by the migratory phase juvenile eel used in this study being highly motivated to move upstream and unlikely to respond in a contrary fashion until the stimulus was sufficiently intense. We are unable to discount the possibility of different physiological mechanisms underpinning the behaviours observed [66], and it is unclear whether some *rejections* may have been an unconditioned reflex stimulated at higher field strengths, rather than being a volitional avoidance behaviour. Nevertheless, aside from the mechanisms a deterrent/guidance system will only be effective if it elicits the desired response (e.g. lateral movement or *rejection*) from the management perspective, and this is likely to change with species, site and application (e.g. barrier versus guidance). Hence, further work is needed to understand the mechanisms which underpin this behavioural variability to improve design criteria.

From the perspective of developing behavioural guidance systems for eel, this study demonstrates that under certain field strengths and pulse frequencies the upstream migrating juvenile life-stage exhibits avoidance to electric fields. Enhanced guidance towards areas with a weaker electric field was achieved at higher field strengths and the lower frequency waveform (e.g. 2 Hz rather than 10 Hz), the latter providing the additional benefit of lower risk of injury and power consumption costs. We recommend further investigation to optimise electric field parameters (e.g. pulse frequencies) and electrode orientation (e.g. 45° to the flow) to continue to improve guidance for juveniles, other life stages of eel (e.g. downstream migrating silver-phase and yellow-phase eel) and species (including those that are group living), under different site conditions (e.g. water conductivities in tidal estuaries) and management strategies (e.g. upstream passage solutions). In particular, we suggest that future research should investigate further an approach based on the marginal gains concept to enhance the effectiveness of existing environmental impact mitigation measures, such as eel passes and physical screens, to provide more efficient and cost-effective hybrid behavioural guidance systems.
